# Dietary glycemic index, glycemic load, and cancer risk: results from the EPIC-Italy study

**DOI:** 10.1038/s41598-017-09498-2

**Published:** 2017-08-29

**Authors:** S. Sieri, C. Agnoli, V. Pala, S. Grioni, F. Brighenti, N. Pellegrini, G. Masala, D. Palli, A. Mattiello, S. Panico, F. Ricceri, F. Fasanelli, G. Frasca, R. Tumino, V. Krogh

**Affiliations:** 1 Epidemiology and Prevention Unit, Fondazione IRCCS Istituto Nazionale dei Tumori, Milan, Italy; 2 Department of Public Health, University of Parma, Parma, Italy; 3 Molecular and Nutritional Epidemiology Unit, ISPO-Cancer Research and Prevention Institute, Florence, Italy; 4 Department of Clinical and Experimental Medicine, Federico II University, Naples, Italy; 5 Department of Clinical and Biological Sciences, University of Turin, Turin, Italy; 6 Unit of Epidemiology, Regional Health Service ASL TO3, Grugliasco, Turin Italy; 7 Unit of Cancer Epidemiology, Department of Medical Sciences, University of Turin, Turin, Italy; 8 Cancer Registry, Department of Medical Prevention, ASP Ragusa, Italy

## Abstract

Factors linked to glucose metabolism are involved in the etiology of several cancers. High glycemic index (GI) or high glycemic load (GL) diets, which chronically raise postprandial blood glucose, may increase cancer risk by affecting insulin-like growth factor. We prospectively investigated cancer risk and dietary GI/GL in the EPIC-Italy cohort. After a median 14.9 years, 5112 incident cancers and 2460 deaths were identified among 45,148 recruited adults. High GI was associated with increased risk of colon and bladder cancer. High GL was associated with: increased risk of colon cancer; increased risk of diabetes-related cancers; and decreased risk of rectal cancer. High intake of carbohydrate from high GI foods was significantly associated with increased risk of colon and diabetes-related cancers, but decreased risk of stomach cancer; whereas high intake of carbohydrates from low GI foods was associated with reduced colon cancer risk. In a Mediterranean population with high and varied carbohydrate intake, carbohydrates that strongly raise postprandial blood glucose may increase colon and bladder cancer risk, while the quantity of carbohydrate consumed may be involved in diabetes-related cancers. Further studies are needed to confirm the opposing effects of high dietary GL on risks of colon and rectal cancers.

## Introduction

Factors linked to glucose metabolism seem to be involved in the etiology of several cancers^1–4^. Consumption of most carbohydrates increases blood glucose and blood insulin, but to varying extents, depending on carbohydrate type and processing, amount consumed, and presence of other nutrients. These variations are captured by the glycemic index (GI)^5^, which ranks carbohydrate foods according to their ability to raise blood glucose levels. High GI foods, like white bread, are rapidly digested and cause a rapid peak in blood glucose. Low GI foods like pulses and pasta, are digested more slowly, prompting a more gradual rise in blood glucose. Glycemic load (GL), the product of a food’s GI and its available carbohydrate content, was introduced to incorporate the effect of the total amount of carbohydrate consumed: it is a measure of total glycemic effect, and is hence an indicator of the insulin demand of the diet.

Several observational studies have investigated associations between dietary GI/GL and risk of different types of cancer, but have produced mixed results. Three meta-analyses − one that investigated only cohort studies^6^, and others that considered both case-control and cohort studies^7, 8^ – found that high GI was associated with increased risk of colorectal cancer. Meta-analyses also found that high GI and GL were weakly associated with increased risk of breast cancer^9^ and diabetes-related cancers^6^, while high GL was associated with increased risk of endometrial cancer^6^. The risks of developing other cancers do not appear to be influenced by dietary GI or GL^6, 7, 10, 11^.

Associations of dietary GI/GL with colorectal and breast cancer have been investigated previously in persons recruited to the Italian section of the European Prospective Investigation into Cancer and Nutrition (EPIC-Italy)^12, 13^. It was found that high GI was significantly associated with increased risk of colorectal cancer^12^, and high dietary GL was significantly associated with increased risk of breast cancer^13^. In the present study we updated the follow-up of the EPIC-Italy cohort in order to assess associations of dietary GI and GL with various types of cancer.

## Results

Characteristics of participants in the upper and lower quintiles of GI and GL are shown in Table 1. Mean dietary GI ranged from 50.0 in the lowest to 57.4 in the highest quintile. GL ranged from 86 g in the lowest to 235 g (glucose equivalents) in the highest. Participants in the highest GI quintile consumed more carbohydrate, especially more carbohydrate from high GI foods, more fiber, and more alcohol, but less fat especially saturated and monounsaturated fat, than those in the lowest GI quintile. Participants in the highest GL quintile consumed more carbohydrate, fiber, fat, alcohol, and energy than those in the lowest quintile. Those in the highest GI and GL quintiles smoked more, and those in the highest GI quintile had a slightly higher BMI than those in the lowest. Participants in the highest GL quintile were younger and more educated than those in the lowest.

**Table 1:** Characteristics ^§^ of study participants in the lower and upper quintiles of energy-adjusted*glycemic index and glycemic load.

	Glycemic index		Glycemic load	
	Quintile 1	Quintile 5	Quintile 1	Quintile 5
N	9,089	9,089	9,089	9,089
Glycemic index	50.0 (0.01)	57.4 (0.02)	52.5 (0.03)	54.4 (0.03)
Glycemic load	132.6 (0.51)	167.8 (0.63)	86.0 (0.17)	235.2 (0.38)
High GI (g/day)	90.0 (0.41)	186.5 (0.81)	72.2 (0.24)	231.2 (0.65)
Low GI (g/day)	177.1 (0.70)	106.0 (0.43)	91.7 (0.30)	202.0 (0.68)
Total carbohydrate (g/day)	266.9 (1.02)	292.5 (1.10)	163.9(0.32)	433.2 (0.69)
Fiber (g/day)	22.3 (0.09)	26.4 (0.14)	15.9 (0.05)	34.7 (0.14)
Total fat (g/day)	92.3 (0.32)	82.1 (0.29)	66.95 (0.21)	112.2 (0.32)
Saturated fat (g/day)	32.5 (0.13)	28.1 (0.11)	23.0 (0.08)	39.5 (0.13)
Monounsaturated fat (g/day)	44.0 (0.16)	38.8 (0.14)	32.2 (0.11)	52.4 (0.16)
Polyunsaturated fat (g/day)	10.6 (0.04)	10.5 (0.04)	7.95 (0.03)	13.8 (0.05)
Alcohol (g/day)	11.3 (0.16)	13.7 (0.19)	10.5 (0.16)	14.9 (0.20)
Energy (kcal/day)	2287.0 (7.42)	2299.6 (7.28)	1568.2 (3.38)	3234.4 (5.81)
Age (years)	50.7 (0.08)	50.8 (0.08)	52.4 (0.08)	48.9 (0.08)
Body mass index (kg/m^2^)	25.9 (0.04)	26.2 (0.04)	26.1 (0.04)	26.1 (0.04)
Physical activity (MET-hours)	1.54 (0.001)	1.53 (0.001)	1.53 (0.001)	1.55 (0.001)
Education (% over 8 years)	50	51	46	53
Smoking (% smokers)	24	31	24	31

^§^Table entries are means, except where indicated. *Energy adjustment by residual method.

Figures in brackets are standard errors.

Dietary GI (Table 2) was not associated with risk of all cancers combined, but high GI was associated with increased risk of colon cancer (HR 1.48, 95%CI 1.09–2.01 highest vs. lowest quintile; *P* trend 0.027) and bladder cancer (HR 1.51, 95%CI 1.01–2.25 highest vs. lowest quintile; *P* trend 0.042). GI was not associated with any other cancer.

**Table 2:** Hazard ratios (HR)^†^ of cancer and mortality in the EPIC-Italy cohort in relation to quintiles of energy-adjusted dietary glycemic index (GI). (5112 cancers, 2460 deaths, median follow-up 14.9 years).

Cancer	*N* cases	1	2		3		4		5		*P* trend^§^
		HR	HR	95% CI	HR	95% CI	HR	95% CI	HR	95% CI	
Tongue	76	1	0.68	(0.34–1.36)	0.86	(0.45–1.64)	0.54	(0.25–1.13)	0.69	(0.35–1.39)	0.230
Stomach	146	1	0.72	(0.44–1.17)	0.90	(0.56–1.43)	0.52	(0.30–0.91)	0.66	(0.40–1.12)	0.065
Colon	441	1	1.21	(0.89–1.66)	1.18	(0.86–1.62)	1.18	(0.86–1.62)	1.48	(1.09–2.01)	0.027
Rectum	102	1	1.58	(0.87–2.86)	1.18	(0.62–2.22)	1.08	(0.56–2.09)	0.90	(0.45–1.79)	0.413
Liver	70	1	0.75	(0.36–1.58)	1.17	(0.60–2.28)	0.71	(0.33–1.52)	0.57	(0.26–1.29)	0.221
Pancreas	117	1	0.84	(0.48–1.46)	0.70	(0.38–1.25)	1.06	(0.62–1.83)	0.85	(0.48–1.52)	0.893
Lung	307	1	0.95	(0.65–1.38)	1.10	(0.75–1.62)	0.82	(0.53–1.27)	0.88	(0.53–1.46)	0.486
Melanoma	194	1	1.68	(1.05–2.69)	1.68	(1.05–2.71)	1.39	(0.84–2.30)	1.51	(0.91–2.48)	0.312
Breast	1362	1	0.91	(0.77–1.07)	1.00	(0.85–1.18)	0.98	(0.82–1.16)	1.00	(0.84–1.19)	0.744
Cervix	53	1	2.86	(1.13–7.29)	0.84	(0.26–2.79)	2.43	(0.91–6.44)	1.99	(0.71–5.55)	0.398
Endometrium	203	1	1.34	(0.89–2.01)	0.77	(0.48–1.23)	0.95	(0.60–1.50)	1.05	(0.67–1.63)	0.597
Ovary	135	1	1.14	(0.70–1.88)	1.02	(0.60–1.71)	0.75	(0.42–1.34)	0.78	(0.44–1.39)	0.180
Prostate	481	1	0.92	(0.68–1.23)	1.10	(0.83–1.46)	1.08	(0.81–1.43)	1.13	(0.84–1.51)	0.235
Bladder	251	1	1.17	(0.76–1.78)	1.08	(0.71–1.66)	1.28	(0.85–1.93)	1.51	(1.01–2.25)	0.042
Kidney	136	1	1.06	(0.66–1.72)	0.62	(0.35–1.10)	0.58	(0.32–1.05)	1.19	(0.72–1.96)	0.759
Meninges	75	1	0.78	(0.34–1.76)	1.37	(0.67–2.81)	1.47	(0.71–3.03)	1.45	(0.69–3.02)	0.125
Brain	95	1	1.33	(0.69–2.57)	1.40	(0.73–2.70)	1.57	(0.82–3.01)	0.99	(0.48–2.04)	0.805
Thyroid	132	1	1.21	(0.72–2.04)	0.65	(0.35–1.21)	1.24	(0.73–2.11)	0.90	(0.50–1.60)	0.783
Lymphomas	106	1	1.13	(0.64–1.98)	0.78	(0.41–1.45)	0.90	(0.49–1.66)	0.92	(0.50–1.70)	0.565
Myeloma	72	1	1.16	(0.56–2.38)	0.85	(0.39–1.86)	0.54	(0.22–1.30)	1.37	(0.68–2.78)	0.829
All cancers combined	5112	1	1.06	(0.97–1.15)	1.04	(0.95–1.14)	1.04	(0.95–1.14)	1.06	(0.97–1.16)	0.332
DRC*	2449	1	1.01	(0.89–1.15)	1.02	(0.90–1.16)	1.04	(0.92–1.19)	1.10	(0.96–1.25)	0.141
Mortality	2460	1	1.07	(0.94–1.22)	1.07	(0.94–1.22)	1.10	(0.97–1.25)	1.06	(0.93–1.20)	0.350

^†^Stratified by food frequency questionnaire and adjusted for sex, education, smoking status, BMI, alcohol intake, fibre intake, saturated fat intake, non-alcohol energy intake and physical activity; ^§^Tests for linear trend were calculated after assigning an ordinal number to each quintile. *Diabetes-related cancers.

Dietary GL (Table 3) was not associated with increased risk of all cancers combined, but high GL was associated with increased risk of colon cancer (HR 1.80, 95%CI 1.18–2.74 highest vs. lowest quintile; *P* trend 0.010), and increased risk of DRCs (HR 1.23, 95% CI 1.03–1.48 highest vs. lowest quintile; *P* trend 0.015), as well as decreased risk of rectal cancer (HR 0.42, 95%CI 0.18–0.98 highest vs. lowest quintile; P trend 0.047).

**Table 3:** Hazard ratios (HR)^†^ of cancer and mortality in the EPIC-Italy cohort in relation to quintiles of energy-adjusted dietary glycemic load (GL). (5112 cancers, 2460 deaths, median follow-up 14.9 years).

Cancer	*N* cases	1	2		3		4		5		*P* trend^§^
		HR	HR	95% CI	HR	95% CI	HR	95% CI	HR	95% CI	
Tongue	76	1	1.21	(0.59–2.50)	0.98	(0.44–2.18)	0.58	(0.23–1.49)	0.77	(0.28–2.15)	0.252
Stomach	146	1	0.88	(0.52–1.49)	0.86	(0.50–1.50)	0.64	(0.34–1.21)	0.55	(0.26–1.16)	0.087
Colon	441	1	1.11	(0.81–1.53)	1.18	(0.84–1.67)	1.27	(0.88–1.84)	1.80	(1.18–2.74)	0.010
Rectum	102	1	0.51	(0.28–0.94)	0.47	(0.24–0.90)	0.45	(0.22–0.93)	0.42	(0.18–0.98)	0.047
Liver	70	1	0.48	(0.19–1.21)	0.82	(0.35–1.94)	1.46	(0.63–3.41)	1.50	(0.54–4.16)	0.111
Pancreas	117	1	0.63	(0.34–1.15)	0.92	(0.51–1.67)	0.74	(0.37–1.46)	0.60	(0.26–1.38)	0.415
Lung	307	1	0.95	(0.65–1.38)	1.10	(0.75–1.62)	0.82	(0.53–1.27)	0.88	(0.53–1.46)	0.486
Melanoma	194	1	1.01	(0.63–1.63)	1.03	(0.62–1.71)	1.32	(0.78–2.25)	1.11	(0.58–2.12)	0.446
Breast	1362	1	1.16	(0.97–1.38)	1.07	(0.87–1.28)	1.19	(0. 97–1.46)	1.14	(0.89–1.46)	0.303
Cervix	53	1	1.54	(0.61–3.87)	1.59	(0.59–4.27)	1.00	(0.31–3.19)	2.00	(0.58–6.85)	0.575
Endometrium	203	1	1.43	(0.90–2.28)	1.17	(0.69–1.98)	1.65	(0.96–2.83)	1.56	(0.81–2.98)	0.176
Ovary	135	1	0.84	(0.49–1.45)	0.84	(0.47–1.51)	0.80	(0.42–1.51)	0.81	(0.38–1.71)	0.580
Prostate	481	1	1.15	(0.85–1.55)	1.18	(0.86–1.64)	1.29	(0.90–1.84)	1.12	(0.72–1.73)	0.444
Bladder	251	1	1.17	(0.77–1.76)	1.22	(0.78–1.90)	1.03	(0.63–1.69)	1.17	(0.67–2.07)	0.816
Kidney	136	1	0.59	(0.34–1.03)	0.84	(0.49–1.46)	0.73	(0.39–1.34)	0.54	(0.25–1.17)	0.289
Meninges	75	1	0.77	(0.36–1.65)	1.17	(0.55–2.50)	0.61	(0.24–1.58)	1.37	(0.51–3.67)	0.744
Brain	95	1	1.38	(0.73–2.61)	0.89	(0.43–1.88)	1.01	(0.46–2.23)	0.74	(0.28–1.94)	0.382
Thyroid	132	1	1.25	(0.70–2.24)	1.00	(0.53–1.90)	1.04	(0.52–2.05)	0.92	(0.41–2.06)	0.632
Lymphomas	106	1	1.47	(0.81–2.65)	0.83	(0.40–1.69)	0.72	(0.32–1.59)	1.19	(0.50–2.81)	0.522
Myeloma	72	1	1.30	(0.55–3.11)	1.09	(0.42–2.81)	2.07	(0.83–5.15)	1.23	(0.40–3.75)	0.408
All cancers combined	5112	1	1.03	(0.94–1.13)	1.05	(0.95–1.15)	1.08	(0.97–1.20)	1.05	(0.93–1.20)	0.272
DRCs*	2449	1	1.08	(0.95–1.24)	1.07	(0.93–1.24)	1.20	(1.03–1.40)	1.23	(1.03–1.48)	0.015
Mortality	2460	1	0.90	(0.79–1.03)	0.98	(0.85–1.12)	0.96	(0.83–1.12)	0.84	(0.70–1.01)	0.298

^†^Stratified by food frequency questionnaire and adjusted for sex, education, smoking status, BMI, alcohol intake, fibre intake, saturated fat intake, non-alcohol energy intake and physical activity; ^§^Tests for linear trend were calculated after assigning an ordinal number to each quintile. *Diabetes-related cancers.

As shown in Table 4, high intake of high GI carbohydrate was associated with decreased risk of stomach cancer (HR 0.51, 95% CI 0.27–0.94 highest vs. lowest quintile; *P* trend 0.045), increased risk of colon cancer (HR 1.71, 95% CI 1.19–2.44 highest vs. lowest quintile; *P* trend 0.004) and increased risk of DRCs (HR 1.23, 95% CI 1.05–1.44 highest vs. lowest quintile; *P* trend 0.011). High intake of carbohydrate from low GI foods was associated with lowered risk of colon cancer (*P* trend 0.032) and mortality (*P* trend 0.029).

**Table 4:** Hazard ratios (HR)^†^ of cancer and mortality in the EPIC-Italy cohort in relation to quintiles of intake of high GI carbohydrate and low GI carbohydrate. (5112 cancers, 2460 deaths, median follow up 14.9 years).

Cancer	*N* cases	Carbo-hydrate	1	2		3		4		5		*P* trend^§^
			HR	HR	95% CI	HR	95% CI	HR	95% CI	HR	95% CI	
Tongue	76	High GI	1	0.75	(0.37–1.53)	0.81	(0.40–1.64)	0.66	(0.31–1.40)	0.59	(0.25–1.36)	0.218
		Low GI	1	1.59	(0.77–3.28)	1.26	(0.58–2.75)	0.99	(0.43–2.30)	1.60	(0.74–3.46)	0.585
Stomach	146	High GI	1	0.63	(0.38–1.05)	0.68	(0.41–1.14)	0.61	(0.36–1.05)	0.51	(0.27–0.94)	0.045
		Low GI	1	1.18	(0.69–2.03)	1.21	(0.70–2.11)	1.10	(0.62–1.94)	1.36	(0.78–2.37)	0.395
Colon	441	High GI	1	1.02	(0.74–1.41)	1.29	(0.94–1.77)	1.18	(0.85–1.66)	1.71	(1.19–2.44)	0.004
		Low GI	1	0.94	(0.71–1.25)	0.86	(0.64–1.16)	0.77	(0.56–1.05)	0.75	(0.54–1.03)	0.032
Rectum	102	High GI	1	1.27	(0.72–2.24)	0.70	(0.36–1.38)	0.84	(0.43–1.62)	0.66	(0.31–1.44)	0.141
		Low GI	1	1.38	(0.74–2.56)	1.37	(0.73–2.58)	1.13	(0.58–2.21)	0.98	(0.48–1.99)	0.774
Liver	70	High GI	1	0.72	(0.33–1.60)	0.88	(0.40–1.93)	0.84	(0.36–1.92)	1.43	(0.60–3.37)	0.441
		Low GI	1	1.09	(0.49–2.45)	1.43	(0.65–3.15)	1.40	(0.62–3.15)	1.41	(0.61–3.28)	0.326
Pancreas	117	High GI	1	0.83	(0.48–1.45)	0.60	(0.32–1.13)	0.96	(0.54–1.72)	0.75	(0.38–1.51)	0.582
		Low GI	1	1.86	(1.04–3.32)	1.67	(0.91–3.07)	0.88	(0.43–1.78)	1.33	(0.68–2.57)	0.732
Lung	307	High GI	1	0.90	(0.63–1.29)	0.80	(0.55–1.17)	0.82	(0.56–1.19)	0.82	(0.54–1.25)	0.304
		Low GI	1	0.89	(0.64–1.27)	0.86	(0.60–1.24)	0.84	(0.58–1.21)	1.00	(0.69–1.44)	0.830
Melanoma	194	High GI	1	1.56	(0.98–2.49)	1.46	(0.89–2.38)	1.48	(0.89–2.47)	1.63	(0.95–2.86)	0.171
		Low GI	1	0.79	(0.51–1.22)	0.83	(0.54–1.29)	0.51	(0.31–0.85)	0.79	(0.50–1.25)	0.118
Breast	1362	High GI	1	1.05	(0.88–1.24)	1.08	(0.90–1.29)	1.05	(0.87–1.27)	1.11	(0.90–1.37)	0.381
		Low GI	1	1.04	(0.88–1.24)	1.07	(0.89–1.27)	1.05	(0.88–1.26)	1.09	(0.91–1.31)	0.401
Cervix	53	High GI	1	0.90	(0.37–2.19)	0.85	(0.33–2.15)	1.26	(0.52–3.08)	1.01	(0.35–2.92)	0.720
		Low GI	1	0.79	(0.33–1.91)	1.10	(0.48–2.51)	0.89	(0.38–2.11)	0.60	(0.23–1.56)	0.421
Endometrium	203	High GI	1	0.96	(0.61–1.50)	1.11	(0.71–1.74)	0.93	(0.57–1.52)	0.98	(0.56–1.71)	0.935
		Low GI	1	1.33	(0.82–2.13)	1.49	(0.93–2.39)	1.15	(0.69–1.90)	1.36	(0.82–2.26)	0.471
Ovary	135	High GI	1	0.88	(0.53–1.45)	0.68	(0.39–1.19)	0.64	(0.35–1.16)	0.81	(0.43–1.53)	0.252
		Low GI	1	1.83	(1.04–3.21)	1.49	(0.82–2.72)	1.45	(0.79–2.67)	1.59	(0.86–2.94)	0.402
Prostate	481	High GI	1	1.00	(0.75–1.33)	0.97	(0.72–1.30)	1.17	(0.86–1.58)	1.07	(0.75–1.52)	0.431
		Low GI	1	1.08	(0.81–1.43)	0.90	(0.67–1.21)	0.93	(0.69–1.26)	1.00	(0.72–1.37)	0.638
Bladder	251	High GI	1	1.35	(0.88–2.07)	1.27	(0.81–1.97)	1.51	(0.98–2.34)	1.44	(0.89–2.34)	0.124
		Low GI	1	0.88	(0.61–1.28)	0.91	(0.62–1.33)	0.87	(0.59–1.29)	0.67	(0.43–1.03)	0.112
Kidney	136	High GI	1	1.10	(0.67–1.81)	0.92	(0.53–1.57)	0.61	(0.33–1.15)	1.02	(0.54–1.92)	0.391
		Low GI	1	0.91	(0.65–1.88)	1.11	(0.65–1.88)	0.93	(0.54–1.63)	0.74	(0.40–1.34)	0.418
Meninges	75	High GI	1	0.97	(0.46–2.02)	0.88	(0.40–1.94)	1.21	(0.56–2.63)	1.53	(0.64–3.64)	0.322
		Low GI	1	0.67	(0.33–1.38)	0.89	(0.45–1.76)	0.54	(0.25–1.17)	0.56	(0.25–1.25)	0.1.37
Brain	95	High GI	1	1.10	(0.57–2.15)	1.07	(0.54–2.14)	1.66	(0.85–3.22)	0.85	(0.37–1.98)	0.654
		Low GI	1	1.49	(0.74–2.99)	2.14	(1.10–4.18)	1.42	(0.69–2.94)	1.05	(0.47–2.31)	0.972
Thyroid	132	High GI	1	0.96	(0.55–1.68)	0.99	(0.56–1.75)	0.72	(0.38–1.34)	0.91	(0.47–1.79)	0.526
		Low GI	1	1.14	(0.64–2.03)	1.19	(0.67–2.14)	1.37	(0.77–2.45)	1.06	(0.56–1.99)	0.651
Lymphomas	106	High GI	1	0.78	(0.43–1.42)	0.93	(0.51–1.69)	0.62	(0.31–1.23)	0.97	(0.48–1.98)	0.656
		Low GI	1	1.12	(0.61–2.05)	0.84	(0.43–1.61)	1.09	(0.58–2.03)	0.79	(0.40–1.58)	0.538
Myeloma	72	High GI	1	0.81	(0.36–1.82)	0.71	(0.30–1.66)	1.14	(0.52–2.48)	1.13	(0.48–2.66)	0.541
		Low GI	1	1.10	(0.53–2.30)	0.83	(0.37–1.88)	1.19	(0.56–2.51)	1.00	(0.46–2.18)	0.926
All cancers combined	5112	High GI	1	1.07	(0.98–1.17)	1.02	(0.93–1.12)	1.03	(0.93–1.13)	1.09	(0.98–1.21)	0.338
		Low GI	1	1.01	(0.92–1.10)	1.02	(0.93–1.12)	0.96	(0.87–1.05)	0.98	(0.89–1.08)	0.399
DRCs*	2449	High GI	1	1.05	(0.93–1.20)	1.11	(0.97–1.27)	1.12	(0.97–1.29)	1.23	(1.05–1.44)	0.011
		Low GI	1	0.98	(0.86–1.12)	1.02	(0.89–1.16)	0.95	(0.83–1.09)	0.96	(0.84–1.11)	0.517
Mortality	2460	High GI	1	1.02	(0.90–1.17)	0.89	(0.77–1.02)	1.03	(0.90–1.18)	1.04	(0.90–1.22)	0.651
		Low GI	1	1.01	(0.89–1.14)	0.97	(0.85–1.10)	0.87	(0.76–1.00)	0.91	(0.79–1.04)	0.029

^†^Stratified by food frequency questionnaire and adjusted for sex, education, smoking status, BMI, alcohol intake, fibre intake, saturated fat intake, non-alcohol energy intake and physical activity; ^§^Tests for linear trend were assessed after assigning an ordinal number to each quintile. *Diabetes-related cancers.

When participants being treated for diabetes, or diagnosed with cancer during the first 6 months of follow-up were excluded, the results did not differ from those cited above and all significant associations remained significant or nearly so. When participants who reported they were dieting were excluded, high dietary GL became associated with increased risk of breast cancer (HR 1.34, 95%CI 1.02–1.76 highest vs. lowest quintile; *P* trend 0.049; data not shown).

When the analyses on GL/GI and risk of colon cancer were stratified by subsite (proximal and distal), GI was significantly associated with distal (HR 1.70, 95%CI 1.08–2.67 highest vs. lowest quintile) but not proximal colon cancer (HR 1.32, 95%CI 0.84–2.10 highest vs. lowest quintile). High intake of high GI carbohydrate was associated with increased risk of distal colon cancer (HR 2.23, 95%CI 1.33–3.74 highest vs. lowest quintile; *P* trend 0.011). The results for GL and low GI carbohydrate did not differ between proximal and distal sites. (data not shown in Tables).

## Discussion

The main findings of our study are that high dietary GI was significantly associated with increased risk of colon and bladder cancer; whereas high dietary GL was significantly associated with increased risk of colon cancer and DRCs (which include colon cancer), but decreased risk of rectal cancer. Furthermore, high carbohydrate intake from high GI foods was significantly associated with increased risk of colon cancer and DRCs, but decreased risk of stomach cancer, whereas high carbohydrate intake from low GI foods was significantly associated with decreased risk of colon cancer.

Our finding that high dietary GI, high dietary GL, and high carbohydrate intake from high GI foods, are associated with increased colon cancer risk is in line with the previous EPIC-Italy study^12^ which found that high dietary GI (but not GL) and high consumption of carbohydrates from high GI foods, were associated with significantly increased colon cancer risk. Like the present study, which considered 122 more colon cancer cases than the previous study, our previous study^12^ also found that high consumption of carbohydrates from low GI foods was associated with lowered colon cancer risk: thus taken together the results of both studies suggest that colon cancer risk depends more on the ability of the carbohydrate foods consumed to raise postprandial blood glucose than the overall quantity of carbohydrate consumed.

An alternative interpretation would be that high consumption of highly refined carbohydrates reduces consumption of carbohydrates from low GI foods and hence also reduces consumption of polyphenols and other antioxidants which may protect against colon cancer^14^.

Several cohort studies have examined associations between dietary GI/GL and risk of colon or colorectal cancer. Most found no association, however two^15, 16^ are in broad agreement with our findings: the George *et al*. study^15^ found that high dietary GI was associated with modestly increased risk of colorectal cancer in men but not women; the Women’s Health Study^16^ found that colorectal cancer risk in women was significantly associated with high GL, while the increased risk associated with high GI was not significant. As regards meta-analyses, one published in 2009 (case-control and cohort studies)^17^ and another in 2012^18^ (cohort studies) found no evidence of links between dietary GI/GL and colorectal cancer. However other meta-analyses of cohort and case-control^7, 8^ and cohort^6^ studies found that high GI, but not GL, was associated with increased risk of colorectal or colon cancer. By contrast, we found that colon cancer risk increased significantly with increasing GL (as well as GI) in agreement with a single meta-analysis published in 2008^19^.

We expected that high dietary GL would increase the risk of both colon and rectal cancer (both are DRCs)^20^ but instead found that high GL was associated with significantly lowered risk of rectal cancer. It is known that etiologic factors for the two cancers differ^21–23^; that colon and rectum derive from different segments of embryonic intestinal tract, and differ in function, pH, and exposure to feces^22, 23^; furthermore levels of several bacterial enzymes involved in the production of mutagenic metabolites vary regionally within the large bowel^24^. Any of these differences could have contributed to the opposing effects of high dietary GL on risks of colon and rectal cancers. We have noted previously that high total antioxidant intake, and high adherence to an index of Mediterranean diet had contrasting associations with risks of colon and rectal cancers in the EPIC-Italy cohort^25, 26^.

As regards bladder cancer, we found that high dietary GI was associated with increased risk of this disease. This is consistent with the finding of a 2013 case-control study^27^ that the highest quartile of dietary GI was associated with significantly increased bladder cancer risk. However, although a meta-analysis^6^ found that risk of DRCs was increased in those with high GI (but not GL), when bladder cancer was considered separately, risk was unaffected by GI. Furthermore, a 2009 cohort study^15^ found no association of dietary GL/GI with bladder cancer risk.

Unexpectedly, we found that high carbohydrate from high GI foods was associated with significantly lowered risk of stomach cancer. This result is not inconsistent with the findings of a cohort study^28^ and a case-control study^29^, both of which found that risk of stomach cancer reduced slightly (not significant) as dietary GI and GL increased. Nevertheless a 2016 meta-analysis that analyzed 2 cohort and 4 case-control studies found no significant association between dietary GI/GL and stomach cancer^11^. It is possible that many people who eventually develop stomach cancer have problems digesting foods years before diagnosis and change their diet to mitigate these problems, thus masking any association between carbohydrate intake and this cancer. Stomach cancer risk has been found to be inversely related to socioeconomic status^30^ which could confound the association with high GI foods. However when we adjusted for a proxy of socioeconomic status, results did not change.

As regards DRCs, we found that high dietary GL was significantly associated with increased risk of developing these cancers. This is broadly consistent the results of a meta-analysis^6^ that evaluated 60 811 patients considered to have a DRC from 36 prospective cohort studies. The study found a ‘modest-to-weak’ (significant) association between high dietary GI (but not GL) and risk of developing a DRC. Although we found no association between DRCs and dietary GI, we did find that high carbohydrate intake from high GI foods was significantly associated with increased risk of DRCs. Taken together these findings suggest that increased risk of DRCs may be conferred not by a carbohydrate-rich diet, but by one rich in rapidly-absorbed carbohydrate.

As regards overall cancer risk, our data indicate no association with dietary GI/GL. As far as we are aware just one cohort study has examined associations between GI/GL and overall cancer risk^15^. This study found that high GI was associated with increased risk of total cancer in men but not women, while high GL was associated with reduced overall cancer risk in both men and women.

For breast cancer, we found that neither GI nor GL had any significant association with the risk of this disease. However a subgroup analysis that excluded participants who reported they were dieting at recruitment found that high GL was significantly associated increased breast cancer risk (HR 1.34, 95%CI 1.02–1.76 highest vs. lowest quintile; P trend 0.049). This result is fully consistent with our previous analysis of breast cancer in EPIC-Italy^13^. This study, which *ab initio* excluded all those who were dieting, also found that high GL was significantly associated with increased risk. The present subgroup analysis involved 507 more breast cancer cases and had approximately three more years of follow-up than our original study^13^. A 2016 meta-analysis of 12 cohort studies found that both high GI and GL were significantly associated with modestly increased risk of breast cancer^9^.

Regarding endometrial cancer, all published meta-analyses^7, 19, 31–34^ report a direct significant association between dietary GL and risk of endometrial cancer. However we found that although the risk of this cancer increased with increasing GL quintiles, the increases were never significant and P trend was 0.176. We only had 203 cases, so the lack of a significant association could be due to insufficient power.

Although several etiological hypotheses have been put forward, chronically high blood glucose giving rise to hyperinsulinemia, insulin resistance, and enhanced bioactivity of the IGF axis (and in particular of the potent mitogen IGF-1) is the most commonly-invoked mechanism to account for associations of a high GI/GL diet with increased cancer risk^1^. Insulin may also influence cancer development by altering sex hormone metabolism^35^. The differing associations of dietary GI and GL with different cancers probably reflect the fact that dietary GI is a measure of glucose availability and is independent of quantity, while dietary GL is a measure of the total glycemic effect, and is hence an indicator of the insulin demand of the diet. Thus, since a high GL diet is more likely than a high GI diet to produce chronically elevated blood glucose and insulin, cancer should depend more on dietary GL than dietary GI^36^. Our findings for DRCs fit this scenario: DRC risk was significantly related to high dietary GL and high carbohydrate intake from high GI foods, but not to high dietary GI. Similarly colon cancer risk was significantly associated with high GL and high carbohydrate intake from high GI foods, but also a high GI diet; by contrast high carbohydrate intake from low GI foods was associated with decreased colon cancer risk.

Strengths of our study are its large sample size, prospective design and complete follow-up. It is also notable for the fact that, compared to previous cohort studies, most GI values were determined specifically on local foods and are likely to be more accurate than values estimated from international food tables. Study limitations are first that the FFQs were not specifically designed to furnish dietary GI and GL, although they were designed to provide estimates of total carbohydrate and total energy intake. Second, we only have one dietary measurement and are unable to estimate long-term dietary intake, giving rise to some misclassification of exposure that would be expected to weaken associations between carbohydrate intake and cancer. Third, GI and GL estimates derived from FFQs may not take account of effects due to consuming mixed dishes, varying meal frequency, varying cooking methods, or chewing habits^37^ that can all influence the postprandial glycemic response. Strong correlations between observed and calculated GI values for component foods in mixed meals were found in one study^38^; however another study found that predicted GIs were 22–50%. larger than directly measured GIs suggesting that and dietary GIs estimated from FFQs may be overestimates^39^.

Fourth, although we adjusted for several dietary and lifestyle factors that could confound the association between dietary GI/GL and cancer, residual confounding remains a possibility. It is also possible that unmeasured or unknown factors may have caused confounding. Fifth, because of multiple comparisons, chance might have played a role in our findings although they are consistent with the findings of previous studies on the same cohort^12, 13^. Finally, we have no information on *Helicobacter pylori* infection and were thus unable to examine whether *H pylori* influenced associations between stomach cancer and dietary GI/GL; furthermore, the analyses for stomach cancer could not be stratified by subsite or histologic type because of the small numbers of cases.

To conclude, this Italian study on a Mediterranean population characterized by traditionally high and varied carbohydrate intake suggests that a high GI diet may increase risk of colon and bladder cancer, while a high GL diet may increase risk colon cancer and DRCs in general, but reduce risk of rectal cancer. Further prospective studies are needed to confirm the opposing effects of high dietary GL on risks of colon and rectal cancer.

## Materials and Methods

### Study population

EPIC is a large European study on diet and cancer. EPIC-Italy enrolled 47,749 adult volunteers (men and women) at five centers: Varese and Turin in northern Italy, Florence in central Italy, and Naples and Ragusa in southern Italy^40^. The design, population, and baseline data collection methods of EPIC-Italy are described elsewhere^40^. Participants completed a food-frequency questionnaire (FFQ) and a lifestyle questionnaire after signing an informed consent form. The lifestyle questionnaire solicited information on education, socioeconomic status, occupation, history of previous illnesses and surgery, lifetime tobacco and alcohol use, and physical activity.

Participants lost to follow-up at baseline (n = 206) or who emigrated (n = 840) (zero follow-up); with missing information on diet (n = 874), anthropometry (n = 355) or lifestyle (n = 16); and with ratio of total energy intake (determined from the questionnaire) to basal metabolic rate at either extreme of the distribution (cut-offs first and last half-percentiles, n = 449) were excluded. Persons lost to baseline/emigrated had similar baseline characteristics to study participants with full follow-up (data not shown). After a median follow-up of 14.9 years, 5112 incident cancers and 2460 deaths were identified among study participants.

### Ethics Statement

The study protocol was approved by the ethics committee at the Azienda Ospedaliera of Florence (Italy). At baseline, participants signed a written informed consent to use clinical data for research. Consent forms were stored with barcode ID for subject identification. The ethics committee approved this consent procedure. The study protocol and informed consent procedure met the requirements of Italian legislation and the Declaration of Helsinki of 1975, as revised in 2008.

### Dietary assessment

Dietary intake during the year before recruitment was assessed by validated FFQs^41^ (separate ones for Naples, Ragusa, and Varese-Turin-Florence) designed to capture local eating habits. Detailed descriptions of the questionnaires are available elsewhere^42^. Nutrient values for all food items in the FFQs were obtained from Italian food composition tables^43^. GIs for about 150 commercially available Italian foods and prepared foods were obtained from published data^44^, while GIs from other foods are available from unpublished data (Brighenti F, University of Parma). If there was no analyzed food item sufficiently similar to a consumed item, GIs published elsewhere (International GI Tables^45^ and www.glycemicindex.com) were used. Detailed descriptions of the procedure for linking FFQ responses to GIs are given elsewhere^46^.

The average dietary GI of each participant was calculated as the sum of the GIs of each food item consumed, multiplied by the average daily amount consumed and the carbohydrate content (percentage), all divided by the total daily carbohydrate intake. Dietary GL was calculated similarly except that there was no division by total carbohydrate intake. Each unit of GL is equivalent to the blood glucose-raising effect of consuming 1 g of glucose.

We divided carbohydrate intake into that from high GI foods and that from low GI foods, adopting GI 57 as cut-off, such that high and low GI foods each contributed 50% of mean total carbohydrate intake in the EPIC-Italy cohort. The main sources of carbohydrate from high GI foods were bread, sugar/honey and jam, pizza, and refined rice; the main sources of carbohydrate from low GI foods were pasta and fruit.

### Follow-up

In Varese, Turin, Florence and Ragusa, incident cancer cases were identified by linkage to regional cancer registries. In Naples, incident cases were identified through linkage to the regional archive of hospital discharges, and by telephone enquiry where necessary. Participants were followed from study entry until first cancer diagnosis (except non-melanoma skin cancer), death, emigration, or end of follow-up, whichever occurred first. Follow-up ended December 31, 2010, in Florence, Turin, Ragusa and Naples; and December 31, 2009, in Varese. This difference was due to the fact that cancer registry and hospital discharge file availability for updating varied with recruitment center. Cancer cases were identified from the codes of the second edition of the International Classification of Diseases for Oncology. Only cancers with over 50 cases were included in cancer site-specific analyses for statistical power reasons. Diabetes-related cancers (DRCs) are cancers of bladder, breast, colon, rectum, endometrium, liver, pancreas and prostate, as identified in the consensus report of the American Diabetes Association and the American Cancer Society^20^. Diabetes is associated with reduced risk of prostate cancer and increased risk of the other diabetes-related cancers^20^. We assessed associations of dietary carbohydrate with DRCs excluding prostate cancer. Data on vital status, cause and date of death were obtained from mortality databases. Causes of death were coded according to the International Classification of Diseases, 10th Revision.

### Statistical methods

Hazard ratios (HRs) of developing cancer in relation to quintiles of carbohydrate from high GI foods, carbohydrate from low GI foods, dietary GI and dietary GL were estimated by Cox multivariate models stratified by FFQ (north-central Italy, Naples and Ragusa) to control for differences in questionnaire design. The quintiles were defined on the whole cohort and the variables were adjusted for total energy intake using the residual method^47^. In all models, age was the primary time variable. Sex, non-alcohol energy intake, smoking (never smoker/former smoker/current smoker), education (years of schooling), alcohol intake (abstainer, <12 g/day, ≥12 g/day and ≥24 g/day), BMI (<25, ≥25 & <30, ≥30), fiber intake (tertiles), saturated fat intake (g/day) and physical activity (quartiles of MET-hours) were included in the models as confounders.

The significance of linear trends was assessed by treating each quintile as a continuous variable in the model and performing the Wald test.

Sensitivity analyses were carried out, first by excluding persons diagnosed during the first six months of follow-up, second by excluding those with diabetes, and finally by excluding those who reported they were dieting. The analyses were performed with STATA (version 14.0; Stata Corp, TX, USA).
